# Dual-Acting Zeta-Potential-Changing Micelles for Optimal Mucus Diffusion and Enhanced Cellular Uptake after Oral Delivery

**DOI:** 10.3390/pharmaceutics13070974

**Published:** 2021-06-27

**Authors:** Ahmad Malkawi, Nasr Alrabadi, Ross Allan Kennedy

**Affiliations:** 1 Department of Pharmaceutical Sciences, Faculty of Pharmacy, Isra University, Queen Alya Airport Street, Amman 11622, Jordan; 2 Department of Pharmacology, Faculty of Medicine, Jordan University of Science and Technology, Irbid 22110, Jordan; nnalrabadi@just.edu.jo; 3 School of Biomedical Sciences, Charles Sturt University, Wagga Wagga, NSW 2650, Australia; rokennedy@csu.edu.au

**Keywords:** nanoemulsions, micelles, SEDDS, zeta potential, sustained release

## Abstract

Context: Overcoming the intestinal mucosal barrier can be a challenge in drug delivery. Nanoemulsions with negative zeta potentials can effectively permeate the mucus layer, but those with positive zeta potentials are better taken up by cells; a nanoemulsion with capricious zeta potential from negative to positive can achieve both good permeation and high uptake. Objective: This study aimed to develop dual-acting zeta-potential-amphoteric micelles enabling optimal muco-permeation and enhancement of cellular uptake. Methods: A micellar pre-concentrate was prepared from 15% Labrasol, 15% Kolliphor EL, 30% Kolliphor RH 40, and 40% dimethylsulfoxide. The micellar pre-concentrate was loaded with anionic stearic acid (SA), forming ionic complexes with cationic polymers at a ratio of 25:1 with Eudragit RS 100 and Eudragit RL 100. Blank micelles and those containing complexes were separately diluted in physiological buffers and examined for their droplet sizes, polydispersity indices (PDIs), zeta potentials, and cytotoxicity. The SA release from the micellar complexes was evaluated in 0.1 mM phosphate buffer (pH 6.8) containing 0.001% fluorescein, thereby enabling an instant decrease in fluorescence. Finally, the micelles were loaded with the model drug fluorescein diacetate (FDA) and evaluated for their muco-permeation behavior and cellular uptake. Results: The micellar dilutions formed micelles at the critical micelle concentration (CMC) of 312 µg/mL and showed a uniform average droplet size of 14.2 nm, with a PDI < 0.1. Micellar dilutions were non-cytotoxic when used at 1:100 in a physiological medium. Micelles loaded with ionic complexes achieved a sustained release of 95.5 ± 3.7% of the SA in 180 min. Moreover, the zeta potential of the complex-loaded micelles shifted from −5.4 to +1.8 mV, whereas the blank micelles showed a stabilized zeta potential of −10 mV. Furthermore, the negatively charged blank and complex-loaded micelles exhibited comparable muco-permeation, with an overall average of 58.2 ± 3.7% diffusion of FDA. The complex-loaded micellar droplets, however, provided a significantly higher cellular uptake of the model drug FDA (2.2-fold, *p* ≤ 0.01) Conclusion: Due to undergoing a shift in zeta potential, the modified micelles significantly enhanced cellular uptake while preserving mucus-permeating properties.

## 1. Introduction

Oral delivery is the most common route of drug delivery and offers several advantages for localized and systemic therapeutics [1,2,3]. However, the intestinal mucosal barriers, which predominantly comprise the mucus gel layer and absorptive mucosa, limit the delivery of many drugs, especially hydrophobic ones [4,5]. Lipid-based nanocarriers allow poorly soluble drugs to penetrate the intestinal mucosa with high stability [6]. Various nanocarrier strategies for overcoming the intestinal mucosal barriers have been proposed in the literature [3]. These primarily rely on modifying the nanocarrier’s composition, mucoactive properties, and surface zeta potential. It is well-known that the mobilization of nanocarriers through the intestinal mucus layer is facilitated by a small droplet size, negative surface charge, and slippery PEGylated composition. Conversely, the large cationic droplets formed by less-stabilizing non-PEGylated compositions are prone to entrapment in the intestinal mucus [7,8].

The underlying absorptive epithelial layer presents another challenge for drug absorption. Mucoactive systems such as mucoactive nanocarriers can prolong the contact of a formulation with the mucosal epithelia, allowing more of the drug to be released, but may not actually enhance the cellular uptake of the droplets [2,9]. Consequently, an adaptive nanocarrier system that addresses the different challenges presented by those different barriers is needed.

In this regard, the droplet’s surface zeta potential is one of the critical properties in determining a nanodroplet’s competency for muco-permeation and cellular internalization through a negatively charged cell membrane. Nanodroplets that have traversed the intestinal mucus layer are taken up by the intestinal cells much more easily when their surface zeta potential changes from negative to positive [8]. The permeation of the mucus by zeta-potential-changing droplets inhibits reverse diffusion, ensuring a sufficient contact time with the underlying absorptive epithelium for cellular uptake [8,10]. The design of effective zeta-potential-changing nanodroplets must preserve the optimized muco-diffusion of the negatively charged droplets to enhance cellular uptake upon the change in zeta potential [11]. To date, zeta-potential-changing systems for overcoming intestinal mucosal barriers, such as self-emulsifying drug-delivery systems (SEDDS), nanoparticles, and micelles, have been based on the cleavage of a covalent bond from negatively charged phosphorylated structures through the action of alkaline phosphatase (AP). The release of the cleaved anionic phosphate substructures provides the desired positive zeta potential changes exhibited by nanodroplets and shows significantly enhanced cellular uptake. However, these nanodroplets need to be pre-incubated with AP, so they can show poor muco-permeation when applied to intestinal mucus [2,5,12].

Therefore, the main focus of this study was to prepare nanoemulsions with increased cellular uptake while preserving mucus penetration without the need for the pre-incubation with materials such as AP. Our approach was based on producing negatively charged nanodroplets that slowly release their negatively charged loads while permeating the mucus. These negatively charged droplets efficiently permeate the mucus and increasingly become positively charged while approaching the intestinal cells. Consequently, the droplets are expected to show significant cellular uptake following this positive shift in charge (zeta potential).

To achieve this aim, this study utilized mixed micelles formed by non-ionic surfactants, including Kolliphor and Labrasol, mixed with the co-solvent dimethylsulfoxide (DMSO) as a method of drug delivery. Micellar droplets with high anionic density were generated by anchoring excess stearic acid (SA) with a hydrophilic carboxylic acid moiety oriented in the hydrophilic shell [13]. To induce a positive shift in micellar zeta potential, excess SA forming hydrophobic ionic complexes with two lipophilic cationic polymers (Eudragit RS 100 and Eudragit RL 100) was formed and incorporated within the micellar hydrophobic core. These polymers were chosen due to their ability to promote the sustained release of their ionically complexed counter-ions from lipid-based nanoemulsions, as reported previously. Additionally, their effective entrapment within nanodroplets predominantly revealed a positive surface charge exhibited by their permanent cationic ammonium-substructures [14]. By the slow release of free and complexed SA from these two micelles, we sought to change their droplet surface zeta potentials gradually via the exhibited charge of the non-releasing cationic polymers. To validate our approach, the hydrophobic model drug fluorescein diacetate (FDA) was loaded in the prepared zeta-potential-changing micellar droplets, and its diffusion and cellular uptake through porcine intestinal mucus were investigated in vitro.

## 2. Materials and Methods

### 2.1. Materials

Stearic acid (SA), fluorescein diacetate (FDA), fluorescein, potassium phosphate dibasic, sodium phosphate monobasic, Kolliphor EL (macrogol glycerol ricinoleate), and Kolliphor RH 40 (macrogol glycerol hydroxyl stearate) were purchased from Sigma-Aldrich (Vienna, Austria). The copolymers containing ethyl acrylate, methyl methacrylate and trimethylammonioethyl methacrylate Eudragit RS 100 (1:2:0.1), and Eudragit RL 100 (1:2:0.2) were gifted by Evonik AG (Darmstadt, Germany). Labrasol was provided by Gattefossé (Paramus, NJ, USA). All the other chemicals and solvents used were of analytical grade and obtained from commercial sources.

### 2.2. Experimental Methods

#### 2.2.1. Preparation of Ionic Complexes

Complexes of SA with Eudragit RS and Eudragit RL according to Table 1 were prepared in DMSO. A 25:1 ratio between the excess carboxylate charge exhibited by SA and Eudragit RS/RL’s cationic groups was used. The Eudragit RS (10 mg/mL) and Eudragit RL (5 mg/mL) solutions were prepared in DMSO and added (as 100 µL) thereafter to 100 µL volumes of DMSO containing 2 mg of SA. The mixtures were then frozen at −80 °C and lyophilized. The prepared complexes with excess unbound SA were investigated for the development of novel micelles. Another set of complexes containing the same amount of SA at a 1:1 ratio with the polymer was prepared as above.

#### 2.2.2. Characterization of Ionic Complexes Using FT-IR

Using a Bruker ALPHA FT-IR instrument (Billerica, MA, USA) with 22 scans, we recorded the IR spectra of the complex derivatives purified by removing excess SA with n-hexane and their counterparts via platinum attenuated total reflection (ATR). The solids were placed on an ATR tip and scanned at 4000–400 cm^−1^ with a 4 cm^−1^ scanning speed to obtain the FT-IR spectra.

#### 2.2.3. Development of Micellar Complexes

Hydrophobic complexes containing excess SA were dissolved in a mixture of surfactants/co-solvent (% *m*/*v*). The complexes were vortexed at 50 °C in Kolliphor RH 40 (30%), Kolliphor EL (15%), Labrasol (15%), and DMSO (40%) until complete dissolution. Final 0.5 mL micellar solutions containing SA complexed with Eudragit RS and RL were then obtained. Afterward, the micellar complex solutions were mono-dispersed at 1:100 in 0.1 M phosphate buffer (pH 6.8) using a vortex, and the size and polydispersity index (PDI) of the micelles were measured after 0, 2, and 4 h of incubation under shaking at 500 rpm (temperature: 37 °C) using a Malvern Zetasizer device (Malvern Instruments, Worcestershire, UK).

#### 2.2.4. Determination of Critical Micelle Concentration

The critical micelle concentrations (CMCs) of the blank and complex-loaded micellar pre-concentrates were determined using a previously described fluorometric technique [15]. Pyrene—acting as a fluorescent probe partition between the aqueous phase and the micellar hydrophobic core—was dissolved at 6 × 10^−7^ M in 0.1 M phosphate buffer (pH 6.8.) Next, the micellar pre-concentrates were diluted across a range of 100–1000 µg/mL in pyrene solution, vortexed, thermomixed at 60 °C for 2 h, and cooled under shaking overnight. The fluorescence excitation of the samples at λ_em_ = 390 nm was measured using a Tecan microplate reader (Tecan infinite 200, Tecan Austria GmbH, Salzburg, Austria). A decimal logarithmic concentration-*versus*-*I_338_/I_333_* plot was generated to determine the CMC. Finally, the corresponding dilutions of the micelles and Kolliphor surfactants were analyzed individually for droplet size using 0.0001–0.1 dilutions in the buffer.

#### 2.2.5. Safety Test

We next examined the cytotoxicity of micellar complexes diluted to 0.5:100, 1:100, and 3:100 in 25 mM HEPES-buffered saline (HBS, pH 7.4) toward Caco-2 cells [7]. Caco-2 cells in 24-well plates at a density of 2.5 × 10^4^ cells per well were supplied with fresh red MEM three times per week, and incubated under 5% CO_2_ at 37 °C. Following two weeks of incubation, the presence of confluent layers of Caco-2 cells within the plate was verified, and the wells were triple washed with HBS. At this point, 500 µL of micellar complex dilutions were added to each test well, and the plates were incubated for 4, 24, and 48 h at 37 °C. The positive and negative control wells contained blank HBS and 0.5% Triton-X 100 in HBS, respectively. At the end of incubation, the wells were washed twice with HBS again, and a resazurin assay was performed by adding 250 µL of 2.2 mM resazurin in HBS to each well. The resazurin-treated plates were incubated for 3 h, and 100 µL aliquots were then withdrawn from each well and checked for fluorescence at excitation/emission wavelengths of 540/590 nm using a microplate reader (Tecan Infinite 200, Tecan Austria GmbH, Salzburg, Austria). The maximum fluorescence intensity of the positive control based on resazurin’s transformation into detectable pink resorufin was used as a reference against which to compare the fluorescence exhibited by the test wells, to calculate the cell viability percentage as per the following equation:

(1) $$Cell\;viability\;\left(\%\right)=\frac{Fluorescence\;of\;test\;wells}{Fluorescence\;of\;positive\;control\;}\times 100.$$

#### 2.2.6. Complex Dissociation and Partition Coefficient

To evaluate the dissociation and partition coefficient, the SA complexes were centrifuged using a MiniSpin device (Eppendorf AG, Hamburg, Germany) in 1 mL of n-hexane until a free SA clear solution was obtained. The supernatants were then removed, and the precipitated complexes were washed with n-hexane. Thereafter, four tubes for each complex labeled with the time points 0.5, 1, 2, and 3 h were shaken at 500 rpm at 37 °C in 1 mL of 1 mM phosphate buffer (pH 6.8), and 10 µL aliquots were added to 980 µL volumes of a fluorescently sensitive quantifying medium: 0.1 mM phosphate buffer (pH 6.8 (containing 0.001% fluorescein)) plus 10 µL of methanol. For the reference, complexes were dissolved in 1 mL of methanol, and 10 µL aliquots were added to 980 µL volumes of quantifying medium plus 10 µL of 1 mM phosphate buffer (pH 6.8). To determine complex dissociation, the values for the test tubes were compared to those for the reference regarding the decrease in fluorescence intensity using excitation/emission wavelengths of 485/515 nm. The complex partition coefficient (*Log P_n-octanol/water_*) was used to dissolve increasing concentrations of complexes in n-octanol and water until clear solutions were obtained according to the following equation:

(2) $$Log\;P_{n-octanol/water}=log\left(\frac{maximum\;solubility\;in\;n-octanol}{maximum\;solubility\;in\;water}\right).$$

#### 2.2.7. Release of Stearic Acid

The quantification of SA having weak acidic properties (p*K*a = 4.75) in the aqueous medium indicated that SA caused a concentration-dependent decrease in the pH, as detected using a pH electrode, as described previously by Washington and Evans [16]. However, this previous method was modified by transforming the pH changes into instrumentally detectable fluorescent changes. For this purpose, 0.5 mg of pH-sensitive fluorescein were dissolved in a falcon tube containing 50 mL of 0.1 mM phosphate buffer, with the pH adjusted to 6.8, and used as a release medium. The sensitivity of the release medium to decreases in pH was verified by adding gradients of acetic acid and octanoic acid, which caused instant decreases in fluorescent intensity due to fluorescein transformation. Thereafter, 100 µL volumes of 1, 0.5, and 0.25% (*m*/*v*) SA solutions in methanol were drawn separately into 15 mL tubes, and, then, the methanol was allowed to evaporate. Different amounts of precipitated SA were reconstituted in 10 mL of the release medium, vortexed, and heated for a few minutes, producing 0.01, 0.005, and 0.0025% (*m*/*v*) SA. By measuring the decreases in fluorescence according to different SA concentrations in the release medium, a calibration curve was generated and used as a reference for quantifying the SA released from micellar dispersions. Based on the recommendations by Washington and Evans [16], the domain of colorimetric changes in the release medium was beyond that of the used concentrations of SA and, therefore, permitted the further quenching of fluorescein when needed at higher concentrations. To assess SA’s micellar release, 100 µL volumes of the micellar pre-concentrates loaded with different SA complexes were separately mixed with the release medium to 1 mL final volumes and gently vortexed. A dialysis tube (FloatA-Lyzer) was used to dialyze the mixtures against the external phase of a 9 mL release medium in a 50 mL tube at 500 rpm at 37 °C using an Eppendorf ThermoMixer C (Hamburg, Germany). Tubes containing micellar dispersions were tightly closed and incubated for 3 h. We ensured that the release medium’s fluorescence intensity was kept constant and considered the effect of blank formulations containing the same polymer ratios as the complexes omitting SA. At predetermined time points every 30 min, an aliquot of 1 mL was drawn from the external phase of each sample and replaced with fresh release medium. The aliquots were centrifuged at 13,400 rpm for 5 min, and the fluorescence of 100 µL sub-aliquots at excitation/emission wavelengths of 485/515 nm was quantified using a microplate reader (Tecan Infinite 200, Tecan Austria GmbH, Salzburg, Austria). The release was determined as a function of the cumulative decline in fluorescence intensity compared to the reference.

#### 2.2.8. Time-Dependent Zeta-Potential Changes

The predicted zeta-potential changes exhibited by micellar droplets after 3 h of SA release were demonstrated via dynamic light scattering utilizing a Zetasizer. Micelles comprising SA-polymer complexes dispersed in an aqueous medium utilizing 1:100 dilutions were vortexed in 10 mM phosphate buffer (pH 6.8) and evaluated for their zeta potentials every 30 min at 37 °C. We assessed the zeta potentials of both blank micelles as pure excipients and those containing the same percentage of polymers used in complexes without SA, which constituted the lower and upper limits of the zeta-potential values, respectively. Furthermore, the tested micellar complex preparations releasing SA that exhibited time-dependent zeta potentials were always within this range. Changes in the zeta potential were continuously evaluated until the zeta-potential values reached a plateau, where they stabilized.

#### 2.2.9. Muco-Permeation Study

The in vitro Transwell diffusion model described previously was used to evaluate the micellar droplets’ abilities to penetrate the intestinal porcine mucus [2]. Porcine mucus was collected from fresh pig intestines bought from a local slaughterhouse and stored on ice. The intestines were longitudinally incised, and intraluminal chyme was discarded. Consistent uniform mucus was then scraped and collected in a 200 mL beaker, and bulky debris was removed. Further purification was carried out by gently agitating 200 mg/mL of the mucus in 0.1 M sodium chloride for 1 h. The supernatant was centrifuged in 50 mL tubes for 2 h, and the precipitated pure mucus pellets were stored at −20 °C until further use.

By utilizing the DMSO portions of the micellar pre-concentrates, the FDA model drug was prepared as a stock solution, and 0.2% was used in micelles. FDA-spiked micellar pre-concentrates, either blank or containing micellar complexes, were diluted to 1:100 in 0.1 M phosphate buffer (pH 6.8). To characterize the Transwell model, inserts (as donor chambers) closed with a filtering membrane with a 3.0 µm pore size (ThinCert cell culture insert, Greiner-Bio One, Austria) and evenly distributed mucus (50 mg per insert) were separately immersed in a 24-well plate filled with 500 µL of phosphate buffer as the acceptor chamber. Micellar dilutions in the buffer medium (250 µL) were added to the mucus surface of each insert in the donor chamber. A micellar dilution of the FDA load passing through the insert-filtering membrane in the absence of mucus was used as the positive control, whereas the micellar dilution omitting FDA and diffusing the mucus was used as the negative control. Another positive control utilized a free-FDA-saturated solution in the buffer to account for diffusion. Over 4 h, the plates were incubated with 70 rpm shaking, and 100 µL aliquots of the medium were withdrawn in triplicate each hour from the acceptor chambers and transferred into 96-well black plates, with a fresh buffer replacement in the acceptor chamber. Subsequently, 10 µL of 5 M NaOH were added to each aliquot, and the plate was incubated for 30 min to allow the hydrolysis of FDA into fluorescein quantifiable at excitation/emission wavelengths of 480/520 nm. The micellar droplet permeation through the mucus was quantified as a function of the cumulative amount of permeated FDA.

#### 2.2.10. Cellular Uptake Study

Pre-concentrates of micellar complexes and blank micelles labeled with FDA were evaluated in vitro for cellular uptake in Caco-2 cells following a previously described method [2]. Caco-2 cells seeded at a concentration of 2.5 × 10^4^ cells/well within 24-well plates were grown as described above. After obtaining confluent layers of Caco-2 cells, the cells were washed twice with 25 mM HEPES-buffered saline (HBS) (pH 7.4); then, 500 µL micellar dilutions (1:1000) in HBS were added to the cells, which were then incubated at 37 °C under 5% CO_2_ for 4 h. Following incubation, another washing step was performed, and 500 µL of HBS were added to all the wells, excluding those used as positive controls. Subsequent cell lysis was mediated by the addition of 200 µL of 2% Triton-X 100 solution in 5 M NaOH with 30 min incubation to hydrolyze the FDA into detectable sodium fluorescein. The positive control of the FDA reference was represented by cells treated with the lysis solution without removing micellar dilutions, whereas the blank buffer treatment represented the negative control. Aliquots from all the treated wells were transferred to a 96-well plate, and the fluorescence intensity was measured using λ_ex_ = 480 nm and λ_em_ = 520 nm. Cellular uptake was then calculated according to the following equation:

(3) $$Cellular\;uptake\;\left(\%\right)=\frac{Fluorescence\;\left(Sample\right)-Fluorescence\;\left(Negative\;control\right)}{Fluorescence\;\left(Reference\right)-Fluorescence\;\left(Negative\;control\right)}\times 100.$$

#### 2.2.11. Statistical Analysis

GraphPad Prism V.7 was utilized to assess the significance of the differences between micelles in the release study (two-way ANOVA) and cellular uptake study (column statistics) with 95% confidence intervals (*p* ≤ 0.05). * *p* ≤ 0.05 was considered significant, ** *p* ≤ 0.01 very significant, and *** *p* ≤ 0.001 highly significant. The indicated values are expressed as the means ± SD (*n* ≥ 3).

## 3. Results and Discussion

### 3.1. Hydrophobic Complexes

To ensure the optimum saturation of the polymeric cationic binding sites, excess anionic moiety from the counter ion SA was bound. The organic phase was the co-solvent DMSO used as the ion-pairing medium. Besides its relatively low dielectric constant of 46.7 (encouraging ionic interactions) [17], DMSO has a basic strength comparable to that of water [18] and allows the acidic dissociation of the SA carboxylic moiety (p*K*a = 4.75). Furthermore, the concurrent advantageous presence of permanently charged ammonium groups from Eudragit RS/RL can boost the overall ion-pairing process [19]. The findings of a previous study highlighted a major role for stabilized ionic complexes in producing beneficial zeta-potential changes in nanoemulsions following dispersion in aqueous media within a noticeable timeframe [12]. Inducing slow changes in the zeta potential of nanoemulsions from negative to positive or vice versa—often attributed to a slow release of either counter ion—can significantly improve several aspects of drug delivery.

### 3.2. Micelle Characterization

**Figure 1:**
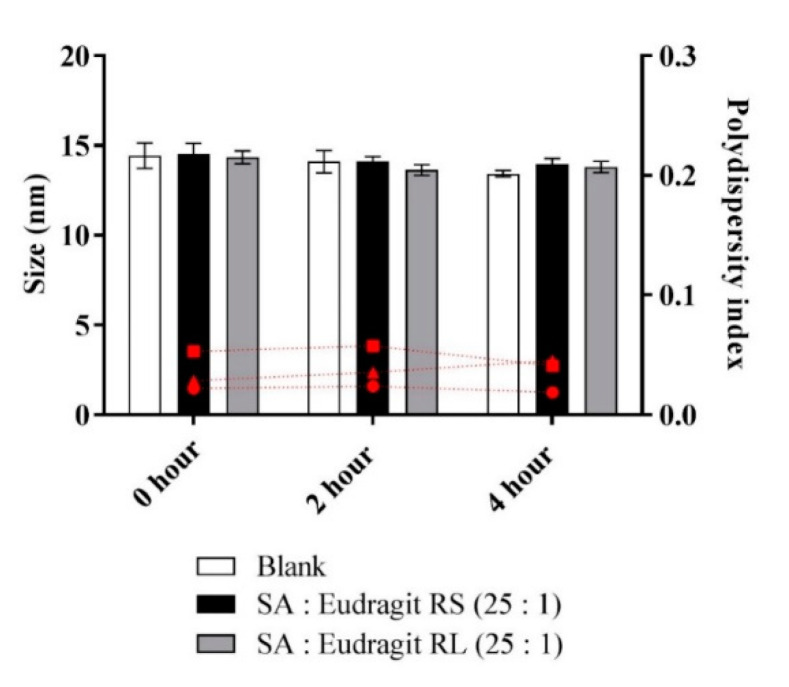
Sizes of micellar droplets used as blank (white bars) or containing SA-polymer complexes with Eudragit RS (black bars) and Eudragit RL (gray bars) and the polydispersity index (red symbols) at 0, 2, and 4 h time points after 1:100 dilution in 0.1 M phosphate buffer (pH 6.8) at 37 °C. Indicated values are the means ± SD (*n* ≥ 3).

The micellar pre-concentrates loading the complexes formed homogenous emulsions when diluted to 1:100 in an aqueous medium. The micellar solutions used as blank or loading complexes were comparable regarding droplet size, exhibiting an average of 14.2 nm, and having a PDI of < 0.1 in an aqueous medium, as illustrated in Figure 1.

Micellar dilutions neither showing phase separation nor precipitation preserved the stability of the size when measured over 4 h. It was possible to determine a stabilized micellar size after ≥ 24 h (data not shown). Furthermore, the fluorometry of the fluorescent probe pyrene in the phosphate buffer at pH 6.8 provided information on the CMC of the diluted micelles. Table 2 highlights similar CMC values for the micelles loading SA-polymer complexes, with the blank micelle showing an average CMC (312 µg/mL).

**Figure 2:**
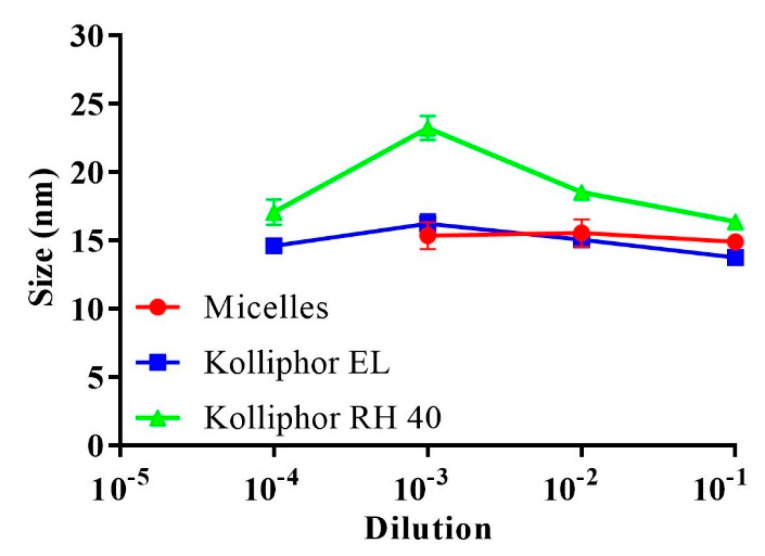
Average droplet size of the micelles and the surfactants Kolliphor EL and Kolliphor RH 40 after 0.1–0.0001 dilutions in 0.1 M phosphate buffer (pH 6.8) at 37 °C. Indicated values are the means ± SD (*n* ≥ 3).

Based on previous observations, altering the hydrophilic-lipophilic balance of the formed surfactant-based micelles towards more lipophilicity reduced their CMC [20]. The concentration of lipophilic complexes from micellar dilutions, however, was too low to alter the self-assembling properties of the diluted surfactants. Moreover, micelles diluted above the CMC before or after the incorporation of complexes exhibited similar stabilized sizes. The corresponding micellar and Kolliphor-based surfactant dilutions in the buffer were examined for droplet size considering the CMC values. As shown in Figure 2, at 0.0001 dilution (<CMC), only the surfactants Kolliphor EL and Kolliphor RH 40 presented an average size of 15.8 ± 1.5 nm, whereas the micellar dilutions showed no measurable size. Above the CMC, dilution at 0.001 showed average sizes of 19.7 ± 3.9 nm and 15.3 ± 0.9 nm for the surfactants and micelles, respectively. Increasing the proportion of polar co-solvents in the micelles interfered with surfactant self-assembly by increasing the CMC [21]. The increased CMC for the present mixture of micellar surfactants compared to that for the individual surfactants in an aqueous medium could be attributed to the use of DMSO as a polar co-solvent. For instance, Anderson et al. noted that the CMC of a cetyltrimethylammonium bromide alkaline solution was shifted from 0.0013 to 0.055 M by using a mixture of water and 60% methanol at 25 °C [22].

Despite the possible impact on the CMC, several advantages were obtained by formulating micellar DMSO. On the one hand, the micellar solubility of the hydrophobic complexes was integrated with the presence of DMSO. On the other hand, DMSO enhanced the miscibility of the relatively small-sized droplets in the surrounding medium. This desired effect facilitated sufficient interactions between the anionic exchange resin anchored within the micellar droplet and the ionic buffer medium to cause dissociation, thereby releasing SA from the micellar complex. Moreover, SA release could be optimized by releasing DMSO while preserving unchanged micellar droplet size, as shown from a previous study [23]. Ultimately, the micellar droplets showed a positive charge in the presence of non-releasing cationic polymers Eudragit.

### 3.3. Cytotoxicity

**Figure 3:**
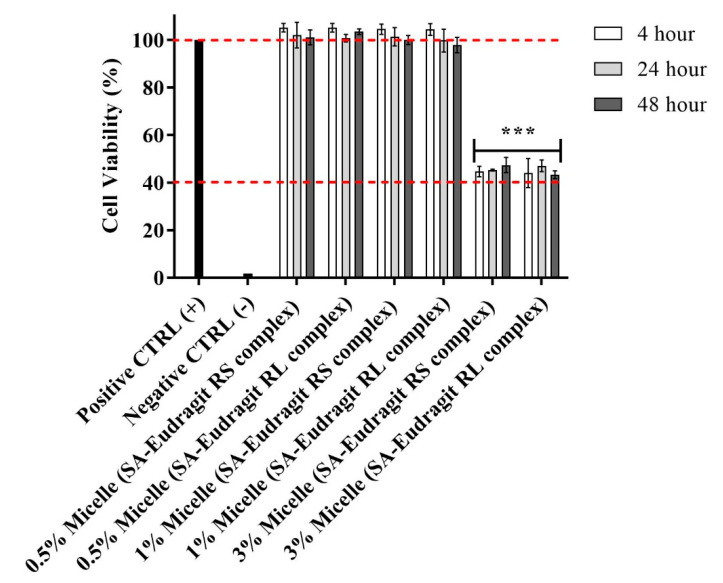
Effects on Caco-2 cell viability of 0.5:100, 1:100, and 3:100 dilutions of micellar pre-concentrates containing SA-polymer complexes with Eudragit RS and Eudragit RL in 25 mM HBS (pH 7.4) after 4, 24, and 48 h incubation at 37 °C. A 3:100 versus 1:100 and 0.5:100 micellar dilutions indicated significant decreases in cell viability (*** p ≤ 0.001). Indicated values are the means ± SD (*n* ≥ 3).

The possible cytotoxic effects of micellar solutions bearing different complex loads were tested using a resazurin assay. To evaluate cytotoxicity, we selected Caco-2 cells, bearing the morphological and functional features of intestinal enterocytes, to test for cytotoxicity, intestinal drug delivery, and cellular uptake after the intended oral administration [24]. As shown in Figure 3, the cell viability remained at 100% under significant micellar concentrations of 0.5% and 1% after 4, 24, and 48 h, providing evidence for prolonged high safety. At a 3% micellar concentration, significant cytotoxicity was observed, as indicated by the 40% cut-off safety limit. Therefore, further studies on cells were conducted using a micellar concentration of ≤1%.

In previous observations, the studied non-ionic surfactants incurred no significant cytotoxicity in micellar compositions at relatively high concentrations [25]. However, a significant increase in the concentration of these surfactants in culture medium was reported to reduce cell viability [26,27]. Furthermore, the cationic properties of polymers, commonly associated with the cell membrane and cellular metabolic disturbances, did not show cytotoxic potential [28]. Several previous evaluations concluded that Eudragit methacrylate copolymers have a high safety limit. Zhang et al., for instance, demonstrated no cytotoxic effects on human cornea epithelial cells after utilizing Eudragit RS, at a concentration of 100 µg/mL, for the coating of a structured lipid-based nanocarrier encapsulating genistein [29]. Therefore, the cytotoxicity induced at a 3% micellar concentration may not be related to the high amount of cationic polymers. Among the micellar excipients, however, DMSO was reported to interact with the cellular membrane and metabolic pathways, causing damages at concentrations > 1% (*v*/*v*) [30]. This could explain why the 0.2% and 0.4% DMSO concentrations from the 0.5:100 and 1:100 micellar dilutions were not cytotoxic, whereas the DMSO concentration of 1.2% from the 3:100 micellar dilution could have an impact on cell viability. Previous studies also reported that DMSO at relatively low concentrations has no cytotoxic potential on different cell lines following 4–48 h incubation [31,32,33].

### 3.4. Dissociation of the Complexes

**Figure 4:**
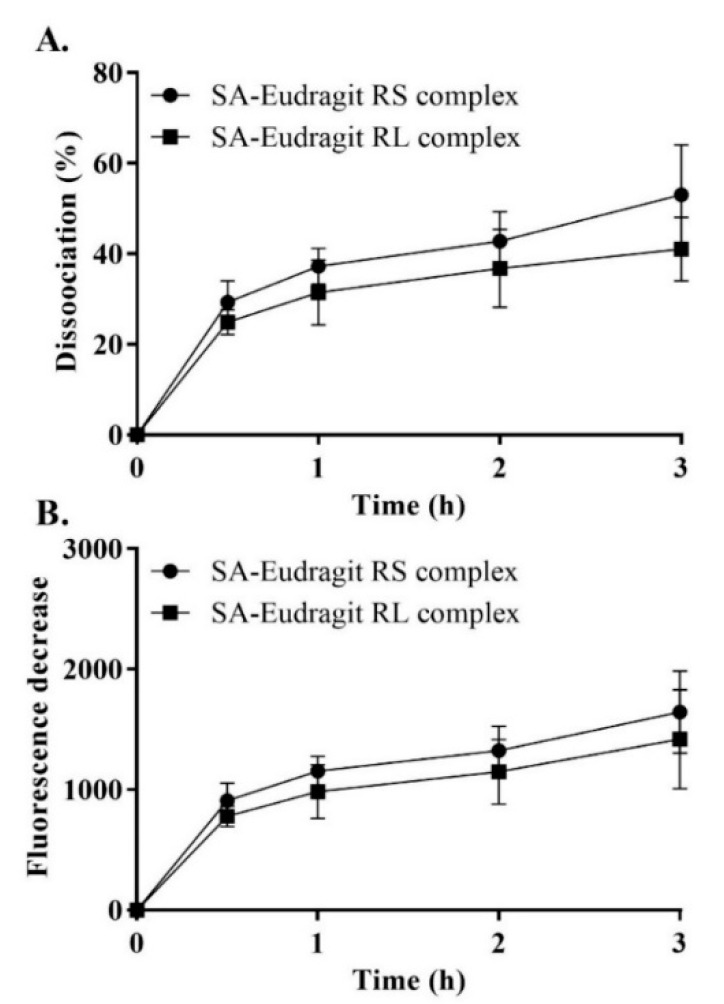
SA–Eudragit RS and SA–Eudragit RL: pure complex dissociation percentage (**A**) and the corresponding fluorescence decrease (**B**) in 1 mM phosphate buffer (pH 6.8) following dilution in a quantifying medium of 0.1 mM phosphate buffer (pH 6.8) containing 0.001% fluorescein at a 1 h time interval for a duration of 3 h. Indicated values are the means ± SD (*n* ≥ 3).

Despite the indispensable need to form stabilized micellar complexes, the reversible nature of ionic interactions is crucial for releasing the counter ion from its micellar complex. Therefore, we examined the potential of SA-polymer complexes to dissociate in a fluorescently sensitive release medium utilizing thermo-mechanical force. For pre-micellar testing, solid dispersions of the complexes showed continuous dissociation of SA each hour, which reached a plateau within 1 h. Over the 3 h dissociation evaluation, up to 47 ± 8.4% of SA rendered polymers as precipitates that dissociated from all the complexes, as shown in Figure 4. A relatively low complex dissociation rate in an aqueous medium was also expected following lipophilic incorporation in the micellar droplets. Our complexes, characterized by the long hydrocarbon SA with insoluble lipophilic Eudragit polymers featuring a tested *Log P_n-octanol/water_* > 5, were found to be sufficiently lipophilic. Moreover, this enhanced lipophilicity ensured the ability to anchor lipophilic complexes within the micellar hydrophobic core, resembling the lipophilicity of the n-octanol phase. Therefore, micelles preserving stabilized complexes over a relatively long period of time were responsible for significant time-dependent changes in the zeta potential throughout the study.

### 3.5. Release Study

**Figure 5:**
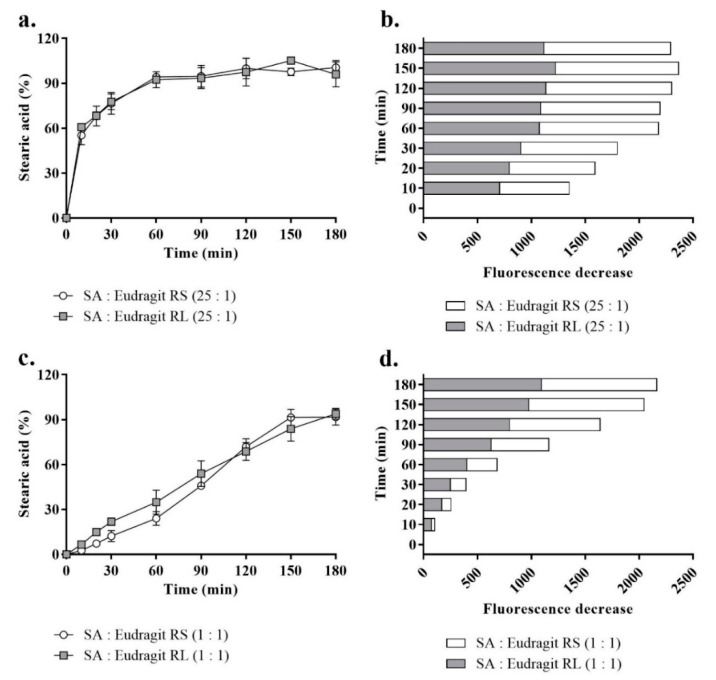
The profiles of SA release from SA-polymer complexes featuring SA-to-polymer ratios of 25:1 with Eudragit RS/RL as a percentage (**a**) and the corresponding fluorescence decrease (**b**) and those of complexes with a SA-to-polymer ratio of 1:1 as a percentage (**c**) and the corresponding fluorescence decrease (**d**) after 1:100 dilution in a release medium of 0.1 mM phosphate buffer (pH 6.8) containing 0.001% fluorescein, taken at 30 min time intervals for 180 min at 37 °C. A 1:1 versus 25:1 SA-to-Eudragit RS/RL ratio led to a significantly greater sustained release of SA (*p ≤* 0.001). Indicated values are the means ± SD (*n* ≥ 3).

The micellar release profiles according to Figure 5 are presented as the released SA percentages corresponding to a fluorescence intensity decrease against time in a fluorescently sensitive aqueous phase at 37 °C. As shown in Figure 5a,b, when SA presented a 25-fold-higher molar equivalence than the micellar Eudragit RS/RL, up to 77.1 ± 5.6% of the released SA (assessed at 30 min) exhibited a rapid release kinetics. As a function of decreasing the pH, Washington and Evans reported a few minutes for the release of >90% free arachidic acid describing a relatively long hydrocarbon fatty acid from submicron emulsion in an aqueous medium [16].

In another study, Torotta et al. also observed the immediate release of the free acidic species indomethacin (log P = 4.27) from micro-emulsions as a function of decreasing pH (monitored with a conventional pH electrode) [34]. Data obtained from these two studies proved that acidic dissociation of poorly soluble acidic species in aqueous media to a large extent enhanced their solubility by showing optimum release. Similarly, as the aim of the present study was to recover a polymeric cationic charge of the micellar droplet by releasing SA, acidic dissociation of free SA was ensured through micellar droplet direct contact with the ionic strength of the aqueous medium. In parallel, the complexed portion of SA showed slower release. The permanently charged ammonium substructures from these two polymers independent of pH formed a sufficiently strong ionic interaction with SA [19,35]. In the subsequent time points, more of the complexed SA released from micelles at a slow rate with > 95% free and complex micellar SA released at 180 min. However, the complexed portion of micellar SA in a ratio of 25:1 to the polymer between time points highlighted insignificant increases in the released SA percentages according to Figure 5a,b. To highlight significant changes in fluorescence decreases (released SA) between the time points, equal amounts of micellar SA were introduced through micellar complexes with the polymers at a 1:1 ratio. Based on this used ratio, the lower portion of Figure 5c,d compared the most consistent SA release profiles representing a significantly sustained release (*p* ≤ 0.001). The release behavior, showing a slow dissociation of the SA complex portion, was crucial for observing a positive shift in the micellar droplet zeta potential caused by the cationic moiety of the complex polymer in a controlled manner. The use of excess SA ensured the longer-term stability of the complexes and favored slower dissociation from the permanently charged non-releasing Eudrgit. Lu et al. described a reciprocal relationship between counter ions in a way that increases the ratio of the model drug to the polymer or vice versa, encouraging longer stability of the other counterpart within nanoemulsions dispersed in a release medium [36].

**Figure 6:**
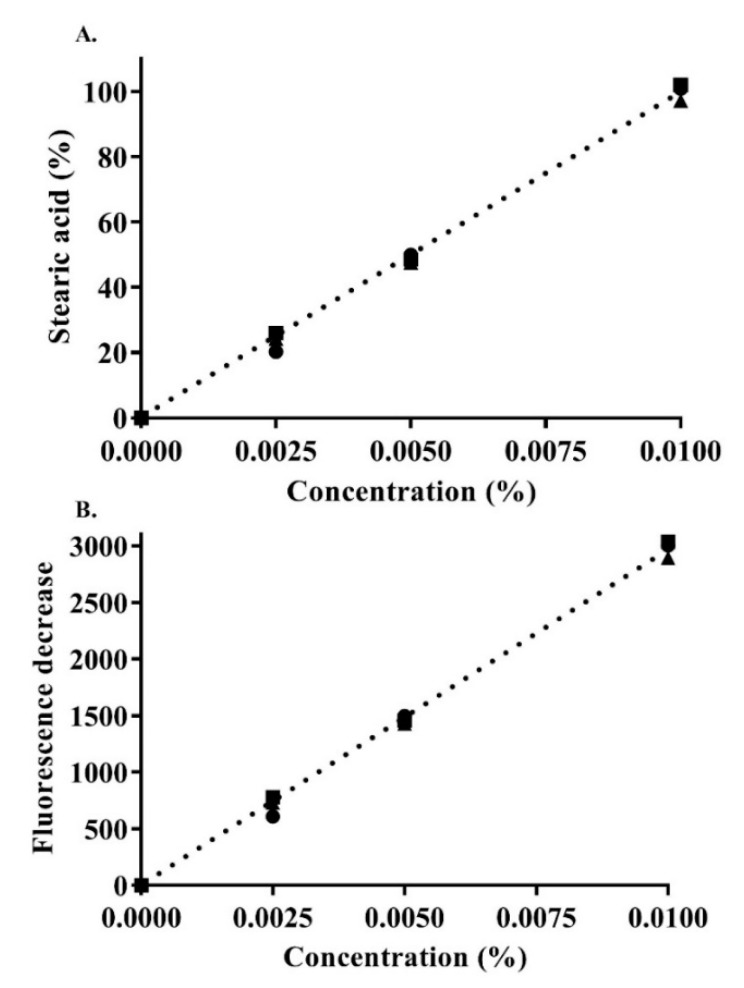
Concentration-dependent SA calibration curve: SA percentage (**A**) as a function of the decrease in fluorescence (**B**) in 0.1 mM phosphate buffer (pH 6.8) containing 0.001% fluorescein. Indicated values are the means ± SD (*n* ≥ 3).

Furthermore, in this study, the ionic strength of the release medium at pH 6.8 with the dissolved fluorescein (0.001%) was reduced in the magnitude of 10^−1^ multiplication. After observing a change in the pH to around ~6.2 at 0.1 mM ionic strength, it was adjusted to 6.8 using sodium hydroxide. This change ensured that the phosphate buffer medium at 0.1 mM would not mask pH changes reflecting the release of SA [16]. Figure 6 shows the calibration curve of the used SA concentrations, indicating linearity with an r^2^ of 0.99 ± 0.002.

At the point that releasing SA stabilized, the release medium verifying > 5 pH indicated further quenching of ionically transformed fluorescein as dianion (phenolic p*K*a ~ 6.5) or monoanion (carboxylic p*K*a ~4) was still feasible with increasing SA (carboxylic p*K*a = 4.75) concentrations [37,38]. To accomplish this, up to 20% acetic acid in a fresh release medium completely vanished any fluorescent properties checked physically and instrumentally after measuring fluorescence. Provided that the p*K*a of releasing SA was not approached in the release medium, quantifying a larger concentration of SA was possible, as it depends on quenching fluorescein demonstrating lower p*K*a. Moreover, SA concentration from the used micellar dilutions having predictable quantification in the release medium in referral to its calibration curve did not form micellar structures and was significantly below its previously reported CMC [13]. Therefore, it can be concluded that the unreached saturation, predictable acid dissociation, and the concentration-dependence of fluorescence sensitivity to SA justified the choice of the release medium [16].

### 3.6. Zeta-Potential Changes

**Figure 7:**
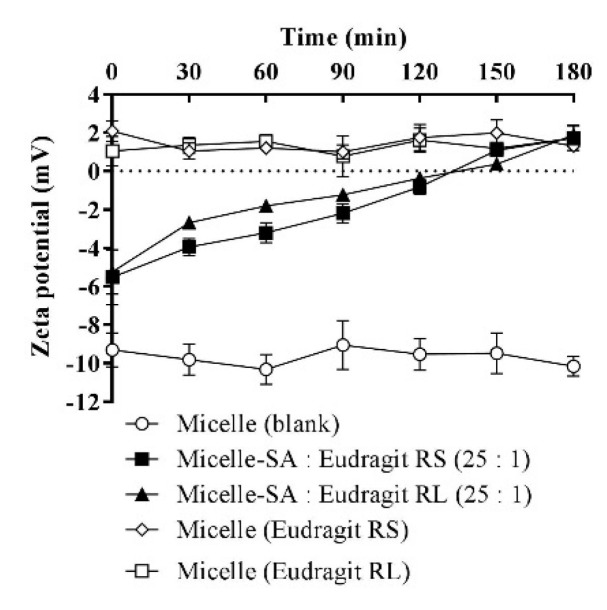
Time-dependent zeta-potential changes of micellar droplets containing SA-polymer complexes featuring a SA-to-polymer ratio of 25:1 with Eudragit RS/RL after 1:100 dilution in 10 mM phosphate buffer (pH 6.8), at 30 min time intervals for 180 min at 37 °C. Zeta-potential values of the blank micelle and micelles containing the same polymer amount used in micellar complexes without SA were also assessed after 1:100 dilution in the buffer. Indicated values are the means ± SD (*n* ≥ 3).

The average zeta potential of SA-polymer complex-loaded micellar droplets at time zero was determined to be −5.4 mV, as shown in Figure 7. Within a 3 h follow-up time, the first positive change in the zeta potential of +0.72 mV was observed for micellar complexes at 150 min, and a further zeta-potential increase to +1.8 mV was minimally observed at 180 min, after which the micellar droplets’ zeta potential stabilized. These zeta-potential values were in the range of −10 to +2 mV, with the lower limit characteristic of a blank micellar dispersion and the upper limit attributed solely to the micellar load of the polymers excluding SA by assuming its full release. The zeta-potential values exhibited by the micelles separately loading these polymers without SA were similar, as both of the polymers were used in charge-equimolar concentrations. Moreover, the use of non-ionic surfactants was necessary. However, further analyses were necessary to refine the surfactant choice to a PEGylated type, such as the Kolliphor type reported to show a negative droplet surface charge [39,40]. Additionally, we observed well-adapted zeta-potential changes corresponding to the added concentrations of cationic polymers dissolved in these micellar surfactants. Therefore, the polymers were adjusted to the lowest possible concentration in the micelles able to provide a positive read and remain masked from excess SA for a considerable duration in the buffer medium. Conversely, the use of SA equivalent from the polymers demanded significantly larger concentrations of the related cationic property that cannot be sufficiently neutralized by the equivalent SA carboxylate. Therefore, micelles loading complexes of equivalent polymeric charge to SA showed significant positive charge transformations in the aqueous medium shortly after dispersion.

### 3.7. Mucus Permeation

**Figure 8:**
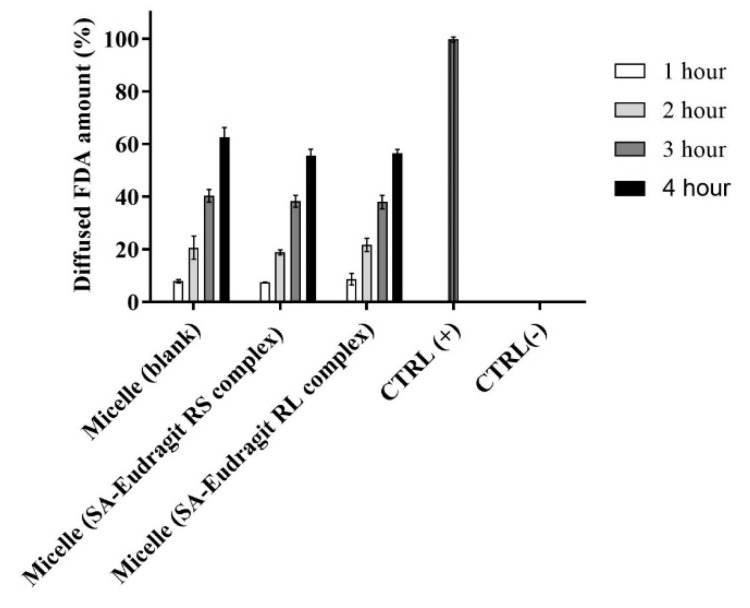
Diffusion behavior of micellar droplets loaded with 0.2% FDA through the intestinal porcine mucus as a blank or containing SA-polymer complexes after 1:100 dilution in 0.1 M phosphate buffer (pH 6.8) for 4 h at 37 °C. Indicated values are the means ± SD (*n* ≥ 3).

The zeta-potential-changing micellar droplets were evaluated in vitro for their diffusion capacity through the intestinal mucus using Transwell diffusion. Figure 8 illustrates dilutions from different micellar complexes and the blank micelle in a 0.1 M buffer (pH 6.80) at 37 °C having an average of 58.2 ± 3.7% of the model drug FDA diffused in the mucus over 4 h.

Generally, several factors must be considered for the efficient muco-permeation of the dispersed nanoemulsion in an aqueous medium, including the droplet size, the droplet surface charge or zeta potential, and the type of excipients forming the nanodroplets [3,41]. The relatively small micellar droplet size was exploited in significantly high muco-permeation of the model drug FDA. The inverse relation between nanoemulsion droplet size and muco-permeation has been described by Friedl et al. In their study, nanoemulsions showing droplet size of 12 nm diffused up to 70% of the model drug through the mucus which was significantly larger than the diffused model drug amount of 8% from relatively large droplets bearing a 455 nm droplet size. They further showed that increasing the proportion of specific excipients such as Kolliphor forming PEGylated corona in aqueous media provides the ultimate flexible and deforming shape of the droplets diffusing the mucus gel layer [42]. Our evaluation utilizing 45% micellar excipients from the Kolliphor source led to efficient muco-permeation. Other research also provided conclusive evidence regarding the aforementioned study [2,43]. Because of droplet size spotting an average below the reported average mucus pore size of 20–200 nm, other factors hindering micellar droplets traversing the mucus could be overcome [41]. On the other hand, the zeta potential exhibited by nanodroplets strongly influences their muco-permeation. The composition of mucin fibers from negatively charged proteoglycans forming the mucus displays selectivity towards the positively charged droplets electrostatically interacting with the mucus, causing major entrapment and poor permeation [8,39]. On the contrary, the diffusion of neutral or particularly negatively charged droplets repelling electrostatic interactions with the mucus is favorable [8]. The amount of zeta-potential-changing micelles penetrating the mucus compared to blank micelle highlighted the insignificant differences. The exhibited negative charge by the zeta-potential-changing micellar droplets lasting sufficiently long helps mitigating interactions with negatively charged mucus components [5]. Therefore, changing the negative zeta potential of studied permeating micellar droplets taking considerable time could explain their similar muco-permeation profiles to that of negatively charged blank micellar droplets not changing the zeta potential. Moreover, shifting the zeta potential of the droplets having already penetrated the mucus to a positive value desirably prolongs their contact time with the underlying absorptive mucosal epithelium for efficient cellular uptake by the means of preventing back-diffusion [10]. It can be concluded that micellar droplets achieved the desired zeta-potential changes while effectively permeating the mucus in a time-dependent manner.

### 3.8. Cellular Uptake

Intestinal absorption by mucosal epithelium accounts for another barrier hindering the mucosal drug delivery of nanoemulsions, especially those exhibiting a negative charge at the droplet surface [44]. As the time for human intestinal mucosal turnover is 4–6 h, nanodroplets shifting the zeta potential to a positive value within this time are desired for cellular uptake [2,45]. Therefore, the achieved positive zeta-potential changes from the studied micellar droplets in 150–180 min (Section 3.6) allowed sufficient time for cellular uptake.

**Figure 9:**
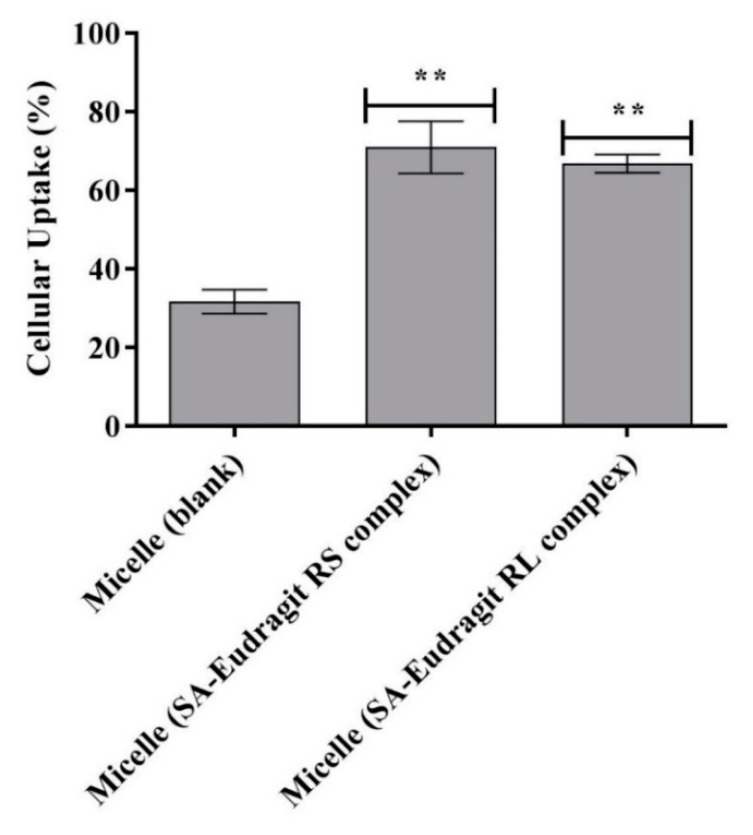
Cellular uptake of FDA-labeled micelles as blank or loaded with SA-polymer complexes after 4 h at 37 °C. ** *p* ≤ 0.01 indicates significant differences regarding SA–Eudragit RS and SA–Eudragit RL complex-loaded micelles versus the blank micelle. Indicated values are the means of at least three experiments ± SD.

Figure 9 illustrates significantly higher Caco-2 cellular uptake of the zeta-potential-changing micelles by ~2.2-fold (*p* ≤ 0.01) from micellar droplets loading SA-Eudragit RS/RL hydrophobic complexes as compared to the blank micelle after 4 h. The permanently negative zeta potential of −10 mV for the blank micelle shifted to a less negative value following SA-polymer complex incorporation and showed further transformation to a positive value upon SA release. This dramatic shift in the zeta potential enhanced micellar droplets’ cellular uptake from 31.7 ± 3% to 68.9 ± 4.9%.

The enhanced cellular uptake of the zeta-potential-changing system has been proven in the literature. The majority of these systems employed cleavable anionic phosphate sub-structures from a backbone lipophilic domain, spiking the zeta-potential-changing nanodroplet by the action of alkaline phosphatase (AP). Applying these nanodroplets having phosphate groups already cleaved in aqueous media, however, was strongly linked to poor permeation of the mucus [2,12,40]. A previous evaluation incorporating 1% of a phosphorylated product of the structure N,N′-Bis (polyoxyethylene) oleylamine in SEDDS showed potent zeta potential change from −15.1 mV to +6.5 mV by the mean of phosphate cleavage following incubation with the AP release medium. This change in zeta potential was desired for enhancing cellular uptake of the AP-pretreated SEDDS but significantly lowered the amount of AP-non-pretreated SEDDS, permeating the mucus to one-third [40]. Moreover, the stringent need of these systems nevertheless to in vitro pretreatment with AP may pose to in vivo variability induced by fluctuations in intestinal enzymatic secretions. Within the study, the zeta-potential-changing micelles releasing SA from regenerated anionic-exchange resins offered a dual action in terms of enhancing cellular uptake after efficient permeation of the mucus. Our approach changing the zeta potential independent of enzymatic activity therefore could be of practical relevance for in vivo applications.

## 4. Conclusions

The blank micelle and the zeta-potential-changing micelles loading anionic SA as hydrophobic ionic complexes with the cationic polymers Eudragit RS and Eudragit RL showed an average CMC of 312 µg/mL, a uniform droplet size of 14.2 nm, and a stability of < 0.1 PDI. Micelles diluted up to 1:100 in physiological medium proved no cytotoxicity on Caco-2 cells. Micellar release combined excess free SA relatively quickly, and SA from the complex was released in an adequately sustained manner. This release pattern caused a significant time-dependent change in the micellar droplet zeta potential, shifting from −5.4 to +1.8 mV. Conversely, blank micellar droplets showed a permanently negative zeta potential of −10 mV. Zeta-potential-changing micellar droplets were shown to be significant, with comparable diffusion behavior between the intestinal porcine mucus and blank micellar droplets. However, they also presented up to a 2.2-fold significantly higher cellular uptake (*p* ≤ 0.01) compared to the blank micelles. Due to the slow and time-dependent changes in zeta potential, micellar droplets, therefore, offer dual benefits in drug delivery by achieving both optimal mucus-permeating properties and significant cellular uptake.

## Figures and Tables

**Table 1:** Hydrophobic ionic complexes of stearic acid with selected cationic polymers.

Stearic Acid	Cationic Polymer	Molar Ratios (Stearic Acid: Polymer)
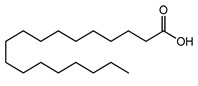 MW = 284.48 g/mol	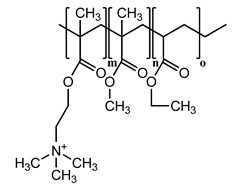 MW = 32 kDa Eudragit RS: m = 0.1, n = 2, o = 1 Eudragit RL: m = 0.2, n = 2, o = 1	25:1 1:1

**Table 2:** Critical micelle concentration (CMC) values of the developed micelles.

Micelle (% *v*/*v*)	Complex	Complex (% *m*/*v*)	CMC (µg/mL)
Labrasol (15%) Kolliphor EL (15%) Kolliphor RH 40 (30%) DMSO (40%)	–	–	314.5 ± 4.8
	SA-Eudragit RS	0.6%	307.8 ± 7.3
	SA-Eudragit RL	0.5%	313.8 ± 4.4

## Data Availability

Not applicable.
